# The role of theory of mind in how increasing preschoolers’ self-esteem affects their materialism: an experimental study

**DOI:** 10.1038/s41598-025-26801-8

**Published:** 2025-11-28

**Authors:** Agata Trzcińska, Wojciech Podsiadłowski, Jowita Wieleszczyk, Ewelina Litwiniec, Aniela Sobuś

**Affiliations:** Faculty of Psychology, University of Warsaw, ul. Banacha 2D, Warsaw, 02-097 Poland

**Keywords:** Materialism, Preschool children, Self-esteem, Theory of mind, Psychology, Human behaviour

## Abstract

This study tested the hypothesis that enhancing preschoolers’ self-esteem in the domains of competence and social acceptance would reduce materialistic tendencies, especially in children with a developed Theory of Mind (ToM). A total of 239 children, 107 girls and 132 boys from Polish preschools participated in an experimental design. Before conducting the main study, a pilot study with 67 preschool children (36 girls and 31 boys) was carried out to validate the effectiveness of the self-esteem manipulation, confirming its success in enhancing children’s state self-esteem in the targeted domains. The results of the main study supported the preregistered hypothesis, showing that self-esteem enhancement reduced materialism but only among children with well-developed ToM. These findings provide insight into how self-perceptions and cognitive abilities, such as ToM, influence materialistic tendencies in young children, highlighting the value of early interventions to foster self-esteem. The results suggest that boosting self-esteem can be an effective strategy for reducing materialistic tendencies in preschoolers, particularly for those with developed Theory of Mind.

**Supplementary Information:**

The online version contains supplementary material available at 10.1038/s41598-025-26801-8.

## Introduction

Materialism, defined as the importance individuals place on possessions as a means of achieving happiness and social status^1^, has become increasingly prevalent, particularly among younger generations^2,3^. According to John’s^4^ model of consumer socialization, preschoolers primarily focus on acquiring more objects, valuing quantity over symbolic meaning. In contrast, recent research suggests that even at this early age, children may already associate material possessions with happiness and success^5^. This variation in the understanding of early childhood materialism highlights the need to examine preschoolers’ materialistic tendencies both as a desire for more material objects (material entitlement) and as a tendency to view possessions as defining their happiness and social status. In line with previous work, children’s materialism^5^ can be understood as a two-dimensional construct encompassing the belief that possessions are central to one’s happiness and serve as indicators of success. The happiness facet reflects the idea that having and acquiring material goods leads to positive emotions and satisfaction, whereas the success facet involves viewing possessions as symbols of achievement and social status. In addition, we distinguish materialism from material entitlement, defined as a sense of deservingness or expectation of receiving material rewards^6^. This conceptual distinction helps to clarify different motivational bases of children’s orientation toward possessions and provides a more nuanced framework for understanding early materialistic tendencies.

Researchers have observed a worrying rise in materialism among children, often linked to negative emotional and social outcomes such as reduced life satisfaction, increased anxiety, and poor emotional well-being^7,8^. Despite the growing interest in understanding the development of materialism in children, much of the existing research has focused on school-aged children and adolescents, with relatively few studies investigating preschool-aged children. Among this younger group, the developmental processes that influence materialism, particularly the role of self-esteem, remain poorly understood. This study seeks to address this gap by experimentally examining how increasing preschoolers’ self-esteem affects their materialistic tendencies, offering an innovative contribution to the literature by exploring a relationship that has, to date, only been examined through correlational research in this age group.

Among children and adolescents, research has shown a clear association between low self-esteem and increased materialism^9,10^. Adolescents and adults who feel insecure or less accepted by their peers often turn to material possessions as a way of signaling their worth or gaining social approval^11,12^. Experimental research with adults and adolescents has further demonstrated that boosting self-esteem can reduce materialism^9,11,13^. However, despite these findings, no experimental studies have been conducted to test whether this relationship holds true for preschool-aged children, where self-esteem is still in its early stages of development. This study, therefore, represents the first experimental attempt to manipulate self-esteem in preschoolers to observe its direct effects on materialism.

In the present study, we conceptualize self-esteem as a dynamic construct that fluctuates in response to situational feedback. Previous researchers^14^ have distinguished between trait self-esteem, referring to a relatively stable evaluation of the self, and state self-esteem, which reflects temporary changes in self-worth arising from specific experiences or social cues. In early childhood, self-perceptions are especially malleable and sensitive to external feedback^15^. Accordingly, the experimental manipulation in this study was designed to elicit increases in state self-esteem.

This study’s focus on self-esteem manipulation is particularly innovative, as it explores two specific domains of self-esteem: perceived competence and social acceptance, as defined by Harter^15^. Preschool children typically evaluate themselves in these two areas - how competent they feel in their cognitive and physical abilities, and how accepted they feel by their peers and caregivers^15^. By experimentally manipulating self-esteem in these domains, this study goes beyond previous research, which has primarily focused on general self-esteem or social self-worth in older children^9^.

Another crucial element of this study is its examination of theory of mind (ToM) as a potential moderator of the relationship between self-esteem and materialism. ToM refers to the ability to understand that others have their own thoughts, beliefs, desires, and emotions, which may differ from one’s own^16,17^. Additionally, some researchers [e.g.,^18^ propose that ToM also involves mentalizing about oneself - reflecting on one’s own mental states and recognizing that they can change over time. Research suggests that ToM is a fundamental cognitive skill that enables children to interpret social interactions and understand how possessions might symbolize status, popularity, or success^19,20^. ToM development also influences how children derive happiness from material goods versus experiences. Chaplin et al.^21^ found that younger children, who have not yet fully developed ToM, tend to derive more happiness from material possessions, whereas older children with more advanced ToM experience greater happiness from social experiences rather than objects. This suggests that as children’s cognitive abilities (particularly ToM) mature, their understanding of the emotional and social value of possessions evolves. However, recent findings suggest that ToM’s role in materialism may be more complex, particularly in the context of self-esteem. Trzcińska et al.^10^ found that preschoolers with more advanced ToM were more likely to exhibit materialistic tendencies when they had low self-esteem, indicating that ToM may not always lead to a reduced focus on possessions. Instead, for children who feel less competent or socially accepted, a well-developed ToM might heighten their awareness of material goods as status symbols and increase their tendency to compare themselves to others, reinforcing materialistic behaviors. However, although Trzcińska et al.‘s^10^ study analyzed the moderating role of ToM in the relationship between preschoolers’ self-esteem and materialism, it was correlational and therefore did not establish causal links.

### Current study

This study aimed to experimentally examine how enhancing self-esteem may influence materialistic tendencies in preschoolers, including both material entitlement and the tendency to treat possessions as defining their happiness and success. We hypothesized that:

H1. *Children with induced high state self-esteem linked to competence (H1a) and social acceptance (H1b) will express lower levels of materialism than children in the control group (no self-esteem prime).*

In preschoolers, the relationship between self-esteem and materialism emerges only when they have a well-developed ToM^10^. Children with more advanced ToM may be more inclined to view possessions as a way to signal social status or self-worth, meaning that a well-developed ToM could strengthen the link between low self-esteem and materialism in preschoolers. Thus, in our experimental study, we hypothesized that:

H2. *The effect of inducing high state self-esteem linked to competence (H2a) and social acceptance (H2b) on materialism levels is moderated by the Theory of Mind*,* indicating that this impact is significant solely among children with well-developed Theory of Mind skills.*

Before conducting the main experimental study, we tested the manipulations in a pilot study.

## Pilot study

To achieve our main research goal, we developed a tool aimed at raising children’s self-esteem. According to Harter^15^, preschool children are generally unable to explicitly articulate global self-esteem. Instead, they express it in two specific areas: (1) perceived competence and (2) perceived social acceptance. Thus, in our study, we focused on manipulating children’s self-esteem within these two domains. To adhere to ethical standards, our manipulation was designed solely to raise (or maintain) children’s self-esteem, with no attempts to lower it. This pilot study aimed to verify whether our experimental manipulation effectively raises children’s state self-esteem.

### Method

#### Participants

We conducted the study in public preschools in a major Polish city. Parents provided consent for their children to participate, and children were also asked for their assent. We recruited 67 children (36 girls and 31 boys) aged 50 to 83 months (*M* = 65.71, *SD* = 7.33).

#### Procedure and experimental manipulation

The study adhered to the principles of the Declaration of Helsinki and received approval from the Research Ethics Committee of the Faculty of Psychology at the University of Warsaw (Opinion Number: 14/11/2023/02). Each child was tested individually in a quiet preschool room by two trained female experimenters, who followed a detailed protocol to ensure procedural consistency across participants. The first experimenter began with the experimental manipulation described below. Each child was randomly assigned to one of three scenarios presented by the first experimenter: (1) a story designed to enhance self-esteem in the area of competence, (2) a story designed to enhance self-esteem in the area of social acceptance, or (3) a control story. Each story was illustrated with simple graphics (clipart images) to help children focus. The clipart illustrations accompanying the stories were prepared in two gender-specific versions so that the characters matched the child’s gender. The images were simple and culturally neutral, and their appropriateness was confirmed in consultation with preschool teachers. To further ensure age-appropriateness, we conducted a small pilot with several preschoolers of similar age, asking them to describe what they saw in the pictures and whether they found them engaging. Feedback from both teachers and children confirmed the suitability of the images used in the study. The content of the individual stories (experimental manipulation) told to the children was as follows:

##### Experimental group 1: inducing high self-esteem linked to competence

“*Recently*,* my colleagues and I were looking at photos of your kindergarten. We noticed that you excel at various tasks there. It is clear that you do well with puzzles*,* coloring books*,* and gymnastic exercises* (the experimenter shows a board with clipart images of children engaged in these activities). *You see*,* after reviewing your kindergarten photos*,* we marked this smiley face* (the experimenter shows the board with smiley faces and points to the happiest one). *This indicates that you are doing exceptionally well with the tasks in kindergarten. I thought it would be nice for you to know this.*”

##### Experimental group 2: inducing high self-esteem linked to peer acceptance

“*Recently*,* my colleagues and I were looking at photos of your kindergarten. We noticed that you have many classmates who want to play with you. It is clear that other children enjoy engaging in games and spending time with you on the playground* (the experimenter shows a board with clipart images of children engaged in these activities). *You see*,* after reviewing your kindergarten photos*,* we marked this smiley face* (the experimenter shows the board with smiley faces and points to the happiest one). *This indicates that you have many friends who enjoy playing with you. I thought it would be nice for you to know this.*”

##### Control group

“*Recently*,* my colleagues and I were looking at photos of your kindergarten. We noticed that you are very cheerful. It is apparent that you often smile and are happy* (the experimenter shows a board with clipart images of children smiling and cheerful but without other children next to them). *You see*,* after reviewing your kindergarten photos*,* we marked this smiley face* (the experimenter shows the board with smiley faces and points to the happiest one). *This indicates that you are usually very cheerful. I thought it would be nice for you to know this.*”

After the experimental manipulation, the first experimenter left the room, and the second experimenter, who was blind to the experimental condition, took over. The second experimenter then measured the child’s mood (controlled variable) followed by state self-esteem. This sequence was identical for all participants.

#### Measures

#####  Self-esteem

The experimenter assessed children’s self-esteem using a Polish adaptation^22^ of Harter and Pike’s Pictorial Scale of Perceived Competence and Social Acceptance for Young Children (PSPCSA) following the instrument’s manual^23^. This tool assesses self-esteem in the domains of perceived cognitive and physical competence (6 items) and perceived social acceptance (peer and maternal, 6 items). The protocol involves presenting children with pairs of pictures and accompanying descriptions of two “types of children” (e.g., “*This boy has a lot of friends to play with*,* this boy does not have a lot of friends to play with. Which one is more similar to you?*”). Children select the picture that resembles them more and indicate whether they are “a lot” or “a little” like that child. Responses are scored on a 1–4 scale, with 1 indicating low perceived competence or acceptance and 4 indicating high. In this study, Cronbach’s alpha was 0.79 for the overall scale, and 0.67 and 0.72 for the competence and acceptance subscales, respectively.

##### Mood (control variable)

The child was asked, “*Before we move on to the next game*,* how are you feeling right now? Which smiley face shows it?*” and presented a board with five smiley faces ranging from sad (1) to happy (5). The child pointed to the face that best represented their mood.

### Results

We conducted a one-way ANOVA to examine differences between the groups, using the Bonferroni adjustment for multiple comparisons. First, we compared the groups on the full scales of perceived competence and perceived acceptance (each consisting of six items). The analysis revealed a significant main effect of the experimental group on perceived competence (*F*(2,64) = 3.586, *p* = .033, partial *η*^*2*^ = 0.101). Children in the competence condition rated their competence higher (*M* = 20.95, *SE* = 0.68) than those in the control group (*M* = 18.55, *SE* = 0.65, *p* = .029). A planned contrast analysis confirmed that this difference was statistically significant, *t*(64) = 2.665, *p* < .05. This result indicates that our manipulation successfully enhanced children’s self-esteem in the area of competence.

However, we found no significant effect of the experimental manipulation on children’s perceived acceptance (*F*(2,63) = 2.419, *p* = .097, partial *η*^*2*^ = 0.071). Nevertheless, it is worth noting that in the domain of acceptance, our experimental manipulation focused solely on peer acceptance, not maternal acceptance. Therefore, we conducted a separate analysis using only the PSPCSA items related to peer acceptance (4 items). This analysis revealed a significant main effect of the experimental group on peer acceptance (*F*(2, 64) = 3.918, *p* = .025, partial *η*^*2*^ = 0.109). Children in the peer acceptance condition rated their peer acceptance higher (*M* = 12.30, *SE* = 0.52) than those in the control group (*M* = 9.91, *SE* = 0.73). A planned contrast analysis confirmed that this difference was statistically significant, *t*(64) = 2.759, *p* < .01. This result indicates that our manipulation effectively enhanced children’s self-esteem in the domain of acceptance, but only in the aspect of peer acceptance.

We also examined whether our experimental manipulation affected children’s mood. A one-way ANOVA indicated no significant differences in mood levels between the experimental groups (*F*(2,64) = 0.237, *p* = .790, partial *η*^*2*^ = 0.007). Thus, we can conclude that our experimental manipulations significantly influenced children’s state self-esteem while having no impact on mood.

### Summary

The experimental manipulation was designed to enhance children’s state self-esteem in specific domains - competence or peer acceptance - by temporarily increasing their perceived standing in those areas. The pilot study confirmed that this procedure effectively raised children’s self-evaluations, and no significant changes in mood were observed, suggesting that it did not simply evoke emotional arousal. These findings support the validity of the procedure as an effective manipulation of state self-esteem.

## Experimental study

### Method

#### Participants

The sample size was determined using G*Power^24^ before conducting the study. We assumed a medium effect size of *f* = 0.25, a significance level of 0.05, and a power of 0.80 in ANOVA with 12 groups, corresponding to three experimental groups and four levels of theory of mind development. Results indicated that 225 participants were needed. The full power analysis protocol is available in the preregistration (OSF). Due to possible exclusions, we aimed to recruit up to 300 children attending public preschools. Children were recruited from seven preschools in Warsaw, Poland, in 2024. To ensure socioeconomic diversity, the study was conducted exclusively in public preschools, as these institutions in Poland typically enroll students from a wide range of social and economic backgrounds. Parents or caregivers provided written informed consent, and only children with parental consent were invited to participate. Before inclusion in the study, children’s verbal assent was also obtained. A total of 242 children were invited to participate. They were informed that they could withdraw from the study at any time, and all children completed the study. However, in accordance with the preregistered exclusion criteria, three children who selected the same response for all items in the materialism measure were excluded from the final analysis. Finally, our sample included 239 children: 107 girls and 132 boys aged 40–92 months (*M* = 67.47, *SD* = 10.94). Of the participants, 231 were Polish, 7 were Ukrainian, and 1 was another nationality; all children spoke Polish.

#### Procedure and experimental manipulation

The study was conducted in accordance with the Declaration of Helsinki and was approved by the Research Ethics Committee of the Faculty of Psychology at the University of Warsaw (Opinion Number: 14/11/2023/02). The study procedure and hypotheses were preregistered on the Open Science Framework (OSF) prior to data collection, https://osf.io/g7e9k/?view_only=60220bb3f189467b8e2f0b655212d11c. Each child participated individually in a quiet preschool room, with two trained female experimenters conducting the sessions. The first experimenter began with a Theory of Mind assessment, followed by the experimental manipulation identical to that in the pilot study. The first experimenter then left the room, and the second experimenter, who was blind to the experimental condition, took over, measuring materialism using the PMT. Afterward, the first experimenter returned, and the second experimenter left. At this point, the first experimenter repeated the experimental manipulation (e.g., *“Do you remember how I told you earlier that I looked at photos from your kindergarten with my colleagues? I would like to remind you of that again. We noticed that you excel at various tasks there*,* such as solving puzzles*,* coloring*,* and gymnastics…”*). The first experimenter then left the room again. Finally, the blind experimenter returned to assess material entitlement. As in the pilot study, the experimenters were trained and followed a detailed protocol to ensure that the procedure was implemented in the same way for all children. The overall sequence of tasks was identical across participants.

#### Measures

#####  Theory of mind

Theory of Mind (ToM) was measured following the procedure used by Chaplin and Norton^25^, using three assessments gauging various aspects of ToM: the Sally and Anne false belief task^26^, the Cookie Box misleading container test^27^, and the Duck and Lion social test^28^. The measures were administered in a rotated order. For each task, children had to respond to a ToM test question (e.g., *“Where will Sally look for her bead?”*) and successfully answer two control questions (e.g., a reality question: *“Where is the bead really?”* and a memory question: *“Where was the bead at the beginning?”*) to earn 1 point. A composite measure was formed by integrating scores from all tasks (ranging from 0 to 3, *KR-20* = 0.59).

#####  Materialism

Materialism was measured in two ways. As the main materialism measure, we administered the Pictorial Materialism Test^5^, incorporating 32 items comprising two equal-length subscales: Happiness and Success. For each item, two images were presented: one representing a materialistic scene (such as having toys or money) and the other illustrating a non-materialistic scene (such as being with a friend or reading with a parent). After hearing descriptions of both images, children were asked to select the picture where the child appeared happier (Happiness Scale, e.g., “*This girl has a lot of friends*,* and this girl has a lot of money. Which girl is happier?*”) or cooler (Success Scale, e.g., “*This boy got a present*,* and this boy paints beautifully. Which boy is cooler?*”). Participants were required to choose just one picture for each item. Materialism scale scores were obtained by totaling the responses, where materialistic options were coded as 1 and non-materialistic options as 0, *KR*-20 was 0.83.

Secondly, we administered the measure of material entitlement. We presented the child with a box containing 20 stickers and asked, “*This is the end of our games together. See*,* here I have stickers. How many stickers do you think you deserve for participating in games with me?*”. The child provided an answer or selected stickers, and the experimenter gave the child as many stickers as chosen. The measure of material entitlement was determined by the number of stickers taken by the child, ranging from 0 to 20.

The data necessary to reproduce the analyses for both the pilot and experimental studies presented in this article are publicly accessible on OSF: https://osf.io/y4au7/?view_only=74cec3bd64a640818b23b5f538d98a1e.

### Results

#### Zero-order correlations

Correlations between ToM, materialism (PMT), material entitlement, age, and sex are presented in Table 1, along with the means and standard deviations.

**Table 1:** Descriptive statistics and zero-order correlations between variables.

Variable	M	SD	1	2	3	4
1. ToM	1.94	1.06	–			
2. Materialism (PMT)	16.27	6.17	− 0.11	–		
3. Material entitlement	2.85	1.91	0.24***	0.13*	–	
4. Age (39–87 months)	67.47	10.94	0.48***	− 0.30***	0.19**	–
5. Sex (0 = female; 1 = male)	–	–	− 0.03	− 0.01	− 0.02	0.01

***p < .001. **p < .01. *p < .05.

#### Hypotheses testing

We hypothesized that children with induced high state self-esteem linked to competence (H1a) and social acceptance (H1b) will express lower level of materialism than children in the control group. Secondly, we hypothesized that the effect of inducing high state self-esteem linked to competence (H2a) and social acceptance (H2b) on materialism levels is moderated by ToM, indicating that this impact is significant solely among children with well-developed ToM. To test our assumptions, we employed 3 (self-esteem prime: competence, social acceptance, control) x 2 (ToM: undeveloped, well-developed) between-subjects ANCOVA models with materialism and material entitlement as dependent variables. We applied the Bonferroni adjustment for multiple comparisons. We categorized the results of ToM into two levels - undeveloped ToM (0–2 points; *n* = 145) and well-developed ToM (3 points; *n* = 94). Such classification was chosen due to the uneven distribution of ToM scores, with only a small number of children exhibiting completely undeveloped ToM. We employed age as the covariate due to its substantial relation with all measures. Exclusion of age as the covariate did not influence the pattern of results presented below (see Supplementary Materials).

We obtained a significant main effect of self-esteem enhancement on materialism, *F*(2, 239) = 6.75, *p* = .001, partial *η*^2^ = 0.055. Participants in the control condition (*M* = 18.08, *SE* = 0.57) reported significantly higher materialism scores than participants in the competence condition (*M* = 15.63, *SE* = 0.71, *p* = .007) and in the social acceptance condition (*M* = 15.20, *SE* = 0.74, *p* = .003). Participants in the latter two conditions did not differ in terms of materialism (*p* = .999).

We did not obtain a significant main effect of self-esteem enhancement on material entitlement, *F*(2, 239) = 2.16, *p* = .117, partial *η*^2^ = 0.018. Participants in the control (*M* = 3.12, *SE* = 0.25), competence (*M* = 2.70, *SE* = 0.19) and social acceptance (*M* = 2.76, *SE* = 0.21) conditions did not differ in material entitlement scores across all comparisons (*ps >* 0.182). Thus, we provided partial support for H1a and H1b with regard to materialism, but not material entitlement.

**Fig. 1:**
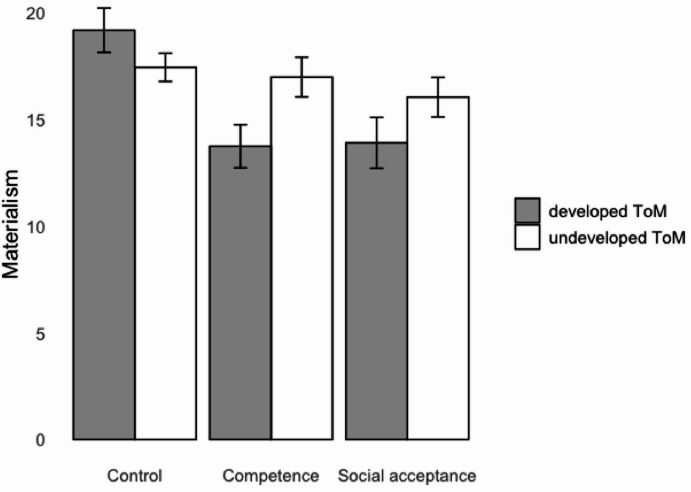
Materialism (PMT) scores for experimental conditions. Error bars show standard errors.

**Fig. 2:**
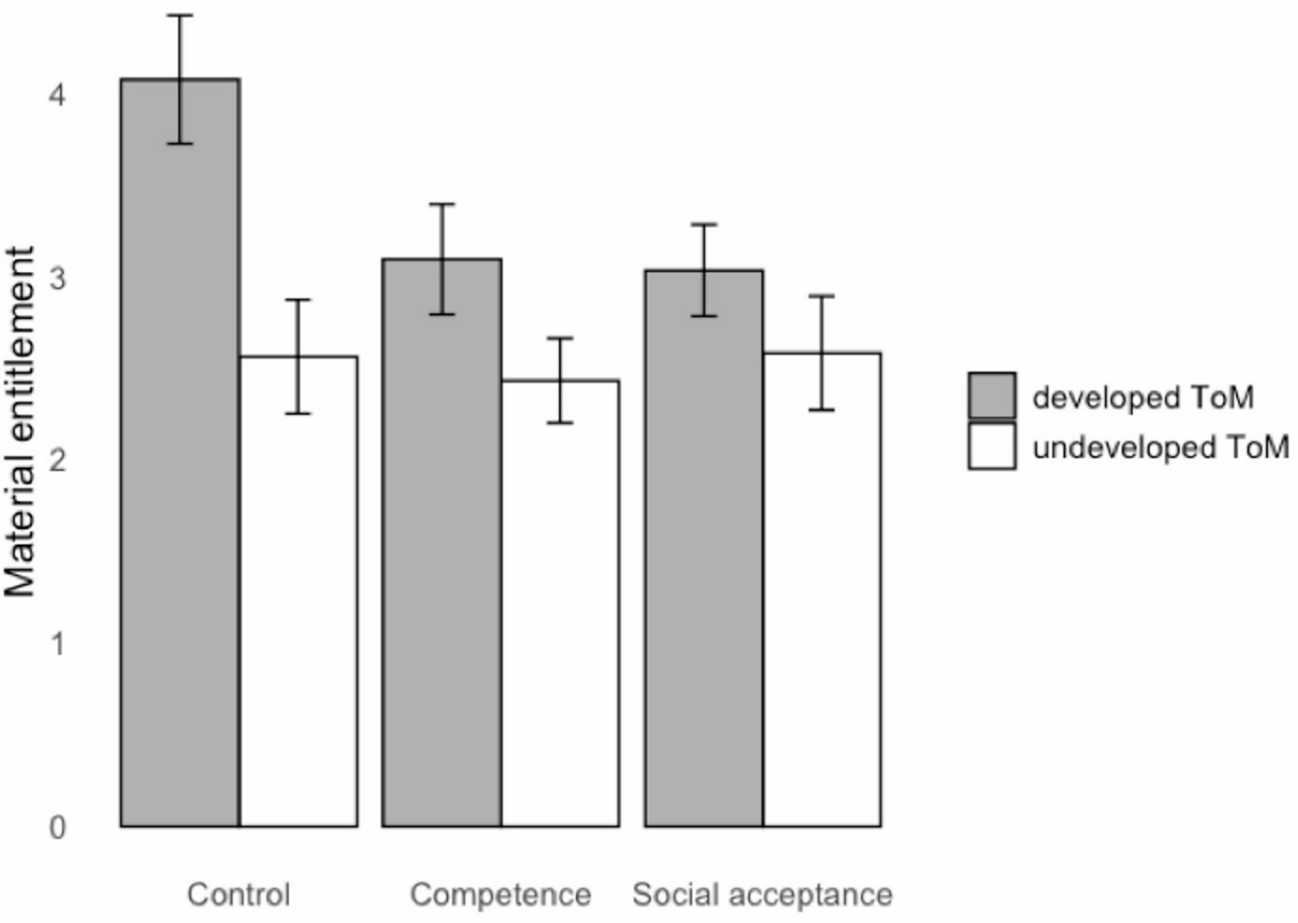
Material entitlement scores for experimental conditions. Error bars show standard errors.

We found a significant interaction effect of the self-esteem enhancement with ToM on materialism, *F*(2, 239) = 5.00, *p* = .007, partial *η*^2^ = 0.041, but not on material entitlement, *F*(2, 239) = 1.60, *p* = .203, partial *η*^2^ = 0.014. Table 2 presents the means and standard errors for the interaction results. Figure 1 illustrates the results for materialism as the dependent variable, while Fig. 2 shows the results for material entitlement as the dependent variable. As predicted, materialism scores did not differ across experimental conditions among children with undeveloped ToM (*p*s > 0.762), while among children with developed ToM, those in control condition had significantly higher materialism score than those in competence (*p* < .001) and social acceptance (*p* = .003) conditions. For material entitlement, scores did not differ across all experimental conditions (*p*s > 0.082). Thus, we provided partial support for H2a and H2b with regard to materialism, but not material entitlement.

**Table 2:** Means and standard errors across conditions.

Dependent variable	Control condition		Competence condition		Social acceptance condition	
	Undeveloped ToM (*n* = 48)	Developed ToM (*n* = 28)	Undeveloped ToM (*n* = 49)	Developed ToM (*n* = 35)	Undeveloped ToM (*n* = 48)	Developed ToM (*n* = 31)
Materialism (PMT)	17.44 (0.66)	19.18 (1.04)	16.98 (0.93)	13.74 (1.01)	16.04 (0.93)	13.90 (1.19)
Material entitlement	2.56 (0.31)	4.07 (0.35)	2.43 (0.23)	3.09 (0.30)	2.58 (0.31)	3.03 (0.25)

In line with our preregistration, the primary analyses described above were conducted using ANOVA/ANCOVA models with categorical ToM variable. To examine whether similar effects would emerge when treating ToM as a continuous variable, we additionally ran moderated regression analyses (see Supplementary Materials). These analyses confirmed the interaction between competence priming and ToM on materialism but did not yield a significant interaction for the social acceptance condition. While generally consistent with the main results, this difference suggests that the moderating role of ToM may be stronger for competence-related self-esteem, or that at preschool age ToM may operate in a more threshold-like manner.

## Discussion

The findings of this study enhance our understanding of the developmental origins of materialistic tendencies in preschool children, shedding light on the moderating role of Theory of Mind in the influence of self-esteem on materialism. Unlike previous correlational research by Trzcińska et al.^10^, this study employed an experimental approach, providing robust evidence of causal relationships. We manipulated children’s state self-esteem in two domains (competence and peer acceptance) and assessed its impact on materialism, with the manipulation’s effectiveness confirmed in a pilot study.

Our results confirmed that inducing high self-esteem - whether in competence (H1a) or peer acceptance (H1b) - reduces materialistic tendencies (i.e., associating possessions with happiness and social status) in preschool children. This aligns with previous research on older children and adults^9,11^, extending the findings to a younger age group. However, self-esteem enhancement did not affect children’s sense of deservingness regarding material rewards (material entitlement). This suggests that increasing children’s self-esteem primarily influences not the quantity of material possessions they desire, but rather their tendency to associate material goods with happiness and success, consistent with the idea that confident children are less likely to rely on possessions as markers of self-worth^12^.

Nevertheless, further analyses showed that self-esteem enhancement proved effective only in children with a well-developed ToM (H2), consistent with the findings of Trzcińska et al.^10^ in their correlational study. Children with a more advanced ToM are likely better able to understand the social meanings of possessions, viewing them as indicators of status or approval. Thus, boosting self-esteem in the domains of competence and peer acceptance can help reduce reliance on material possessions as happiness and status symbols for these children.

Despite these important insights, there are limitations to consider. Our sample was drawn from a single geographical location, which may limit the generalizability of the findings. Future research should include more diverse populations to examine whether these effects hold across different cultural contexts. Additionally, while we focused on self-esteem in the domains of competence and social acceptance (which are relevant at preschool age^15^, other domains, such as physical appearance or behavioral conduct, also become relevant as children enter primary school. Future studies could explore whether these domains influence materialism as well. Another limitation of this study is the relatively low internal consistency of the Theory of Mind composite score (*KR-*20 = 0.59). This is likely due to the short scale, which included only three tasks. Prior research with preschoolers has shown that individual ToM tasks often have modest reliability, and even composite scores may not reach high internal consistency^29^. Thus, our result is consistent with what is commonly observed in this age group. Nevertheless, the limited reliability may have attenuated the strength of the observed interaction effects. Future studies should consider using a broader set of ToM tasks to obtain a more precise estimate of preschoolers’ theory of mind development. A further issue relates to the nature of the self-esteem manipulation. In the present study, we focused on state self-esteem, which reflects temporary, situational changes in self-evaluation. Although this approach is developmentally appropriate for preschoolers, whose global self-concept is still emerging^15^, it remains unclear how long the observed effects persist and whether similar relations with materialism would be found for trait self-esteem, representing a more stable disposition. Future research should therefore examine the durability of these effects and investigate whether comparable patterns emerge when considering individual differences in trait self-esteem.

This study also has practical implications for early childhood education. The findings suggest that supporting children’s sense of competence and peer acceptance may help reduce the tendency to associate possessions with happiness and status, particularly among children with more advanced Theory of Mind. In preschool settings, this could involve giving children positive feedback, encouraging cooperative play, and emphasizing recognition of effort and strengths rather than relying on material rewards. These implications should, however, be considered preliminary. It is not yet clear whether such effects are lasting, and future studies should examine if classroom-based approaches of this kind can produce stable, long-term changes in children’s attitudes toward material possessions.

In conclusion, our study provides experimental evidence supporting the influence of state self-esteem on materialism in preschool-aged children. Our findings enhance the understanding of how children’s self-perceptions and cognitive abilities (particularly ToM) shape materialistic tendencies, emphasizing the importance of early interventions that promote healthy self-esteem. The results suggest that interventions aimed at boosting state self-esteem can effectively reduce materialistic tendencies in young children, provided they have developed Theory of Mind.

## Supplementary Information

Below is the link to the electronic supplementary material.

Supplementary Material 1

## Data Availability

The data necessary to reproduce the analyses presented here are publicly accessible in the Open Science Framework (OSF): [https://osf.io/y4au7/](https:/osf.io/y4au7).
