# Supplementary material for: The role of theory of mind in how increasing preschoolers’ self-esteem affects their materialism: an experimental study
Source: Sci Rep. 2025 Nov 28;15:42619. doi: 10.1038/s41598-025-26801-8 (PMC12663213; doi:10.1038/s41598-025-26801-8)
Supplement: Supplementary file 1 — Supplementary Material 1 [file 41598_2025_26801_MOESM1_ESM.docx]

**SUPPLEMENTARY MATERIALS**

**ANOVA analyzes**

We obtained a significant main effect of the self-esteem prime on the materialism, *F*(2, 239) = 6.78, *p* = .001, partial *η*^2^ = .055. Participants in the control condition (*M* = 18.08, *SE* = 0.57) reported significantly higher scores of materialism than participants in the competence condition (*M* = 15.63, *SE* = 0.71, *p* = .008) and in the social acceptance condition (*M* = 15.20, *SE* = 0.74, *p* = .003). Participants in the latter two conditions did not differ in terms of materialism (*p* = .999).

We did not obtain a significant main effect of the self-esteem prime on the material entitlement, *F*(2, 239) = 2.02, *p* = .135, partial *η*^2^ = .018. Participants in the control (*M* = 3.12, *SE* = 0.25), competence (*M* = 2.70, *SE* = 0.19) and social acceptance (*M* = 2.76, *SE* = 0.21) conditions did not differ in material entitlement scores across all comparisons (*ps >* .197). Thus, we provided partial support to H1a and H1b with regard to materialism, but not material entitlement.

We obtained a significant interaction effect of the self-esteem prime with ToM on the materialism, *F*(2, 239) = 3.54, *p* = .031, partial *η*^2^ = .029, but not on the material entitlement, *F*(2, 239) = 1.65, *p* = .195, partial *η*^2^ = .014. As predicted, materialism scores did not differ across experimental conditions among children with undeveloped ToM (*p*s > .761), while among children with developed ToM, those in control condition had significantly lower materialism score than those in competence (*p* = .001) and social acceptance (*p* = .003) conditions. When it comes to material entitlement, it did not differ across all experimental conditions (*p*s > .100). Thus, we provided partial support to H2a and H2b with regard to materialism, but not material entitlement.

**Moderated regression analyses**

To investigate the moderating effects of theory of mind treated as a continuous variable (using its full scale from 0 to 3), we employed moderated regression analyses using Model 2 in the Process macro (Hayes, 2022) for SPSS. As in the main text, we used age as a covariate in analyses. Although such a configuration employs theory of mind as the independent variable, and experimental conditions (represented by two binary variables) as moderators, they are statistically equivalent to experimental groups moderated by theory of mind. First, we analyzed the model with materialism as the dependent variable (Table S1). Experimental conditions did not affect materialism independently. However, the interaction of the competence condition and theory of mind was negative and significant, meaning that the negative effect of condition on materialism was stronger among those children who had a more developed theory of mind. The interaction of the social acceptance condition and theory of mind was not significant. Moreover, theory of mind predicted materialism positively, while age - negatively.

**Table S1**

*Results of Moderated Regression Predicting Materialism*

| Variable | *B* | *SE* | 95% *CI* |
| --- | --- | --- | --- |
| Competence condition | 2.24 | 1.83 | [-1.38, 5.81] |
| Social acceptance condition | -0.22 | 1.95 | [-4.05, 3.62] |
| Theory of mind | 1.66** | 0.63 | [0.41, 2.90] |
| Competence condition x Theory of mind | -2.37** | 0.84 | [-4.01, -0.72] |
| Social acceptance condition x Theory of mind | -1.34 | 0.89 | [-3.09, 0.41] |
| Age | -0.19*** | 0.04 | [-0.27, -0.11] |
| *F* | *F*(6, 232) = 7.27*** | | |
| *R*^2^ | .16 | | |

*Note*. Levels of significance: ** *p* < .01, *** *p* < .001.

Second, we analyzed the model with material entitlement as the dependent variable (Table S2). Experimental conditions did not affect material entitlement, nor their interactions with theory of mind and age. The only significant predictor of material entitlement was theory of mind.

**Table S1**

*Results of Moderated Regression Predicting Material Entitlement*

| Variable | *B* | *SE* | 95% *CI* |
| --- | --- | --- | --- |
| Competence condition | -0.10 | 0.59 | [-1.27, 1.07] |
| Social acceptance condition | -0.41 | 0.63 | [-1.66, 0.83] |
| Theory of mind | 0.45* | 0.21 | [0.05, 0.86] |
| Competence condition x Theory of mind | -0.20 | 0.27 | [-0.74, 0.33] |
| Social acceptance condition x Theory of mind | -0.03 | 0.29 | [-0.59, 0.54] |
| Age | 0.02 | 0.01 | [-0.01, 0.04] |
| *F* | *F*(6, 232) = 3.36** | | |
| *R*^2^ | .08 | | |

*Note*. Levels of significance: * *p* < .05, ** *p* < .01.
